# *Drosophila* Ribosomal Protein Mutants Control Tissue Growth Non-Autonomously via Effects on the Prothoracic Gland and Ecdysone

**DOI:** 10.1371/journal.pgen.1002408

**Published:** 2011-12-15

**Authors:** Jane I. Lin, Naomi C. Mitchell, Marina Kalcina, Elly Tchoubrieva, Mary J. Stewart, Steven J. Marygold, Cherryl D. Walker, George Thomas, Sally J. Leevers, Richard B. Pearson, Leonie M. Quinn, Ross D. Hannan

**Affiliations:** 1 Peter MacCallum Cancer Centre, East Melbourne, Australia; 2 Department of Biochemistry and Molecular Biology, University of Melbourne, Parkville, Australia; 3 Department of Anatomy and Cell Biology, University of Melbourne, Parkville, Australia; 4 Department of Biological Sciences, North Dakota State University, Fargo, North Dakota, United States of America; 5 Growth Regulation Laboratory, Cancer Research UK London Research Institute, London, United Kingdom; 6 University of Cincinnati Medical Center, Cincinnati, Ohio, United States of America; 7 Department of Biochemistry and Cell Biology, Monash University, Clayton, Australia; 8 School of Biomedical Sciences, The University of Queensland, Brisbane, Australia; Harvard Medical School, Howard Hughes Medical Institute, United States of America

## Abstract

The ribosome is critical for all aspects of cell growth due to its essential role in protein synthesis. Paradoxically, many Ribosomal proteins (Rps) act as tumour suppressors in *Drosophila* and vertebrates. To examine how reductions in *Rps* could lead to tissue overgrowth, we took advantage of the observation that an *RpS6* mutant dominantly suppresses the small rough eye phenotype in a *cyclin E* hypomorphic mutant (*cycE^JP^*). We demonstrated that the suppression of *cycE^JP^* by the *RpS6* mutant is not a consequence of restoring CycE protein levels or activity in the eye imaginal tissue. Rather, the use of *UAS-RpS6* RNAi transgenics revealed that the suppression of *cycE^JP^* is exerted via a mechanism extrinsic to the eye, whereby reduced Rp levels in the prothoracic gland decreases the activity of ecdysone, the steroid hormone, delaying developmental timing and hence allowing time for tissue and organ overgrowth. These data provide for the first time a rationale to explain the counter-intuitive organ overgrowth phenotypes observed for certain members of the *Minute* class of *Drosophila Rp* mutants. They also demonstrate how *Rp* mutants can affect growth and development cell non-autonomously.

## Author Summary

Ribosomes are required for protein synthesis, which is essential for cell growth and division, thus mutations that reduce *Rp* expression would be expected to limit cell growth. Paradoxically, heterozygous deletion or mutation of certain *Rps* can actually promote growth and proliferation and in some cases bestow predisposition to cancer. The underlying mechanism(s) behind these unexpected overgrowth phenotypes despite impairment of ribosome biogenesis has remained obscure. We have addressed this question using the power of *Drosophila* genetics, taking advantage of our observation that four different *Rp* mutants, or *Minutes*, are able to suppress a small rough eye phenotype associated with a mutation of the essential controller of cell proliferation *cyclin E (cycE^JP^)*. Our findings demonstrate that suppression of *cycE^JP^* by the *RpS6* mutant is exerted via a tissue non-autonomous mechanism whereby reduced *Rp* in the prothoracic gland decreases activity of the steroid hormone ecdysone, delaying development and hence allowing time for compensatory growth. These data provide for the first time a rationale to explain the counter-intuitive organ overgrowth phenotypes observed for certain *Drosophila Minutes*. Our findings also have implications for the effect of *Rp* mutants on endocrine related control of tissue growth in higher organisms.

## Introduction

One of the early phenotypic classes identified in *Drosophila* was the *Minutes*, which were classified based on the heterozygous adults having short slender bristles on the body, a generally smaller body size and a delay in the onset of metamorphosis [1]. It has long been considered that understanding the basis for these phenotypes will provide fundamental clues to the mechanisms underlying the control of cell growth and proliferation as well as of tissue and organ size [2]. In 1976 it became apparent that many *Minute* genes encode Ribosomal proteins (*Rps*) [3] and by 2007 most of the *Minutes* were confidently ascribed to the *Rp* genes [4]. In all organisms, Rps are essential for the assembly and optimal functioning of the ribosome and are, therefore, obligate for protein synthesis and cell growth (reviewed in [5]–[6]). Due to their essential role in ribosome biogenesis, mutations that reduce *Rp* expression would be expected to limit cell growth. This cell intrinsic requirement for Rps explains many aspects of the *Minute* phenotype, such as the thin bristles and reduced body size in some *Minutes*. In contrast, other aspects of the *Minute* phenotype have remained enigmatic.

Paradoxically, reduced levels of some *Drosophila* Rps result in overgrowth of specific tissues. For example, *RpS6* mutant larvae have overgrown lymph glands, due to increased growth and over-proliferation of the lymph gland cells [7], and develop melanotic masses [8]–[9], a characteristic feature of over-proliferation of hemocytes [10]. Thus reduced *RpS6* expression results in tissue overgrowth, consistent with *RpS6* having a tumour suppressor like function. Similarly, we have shown that *RpL5* or *RpL38* heterozygous adult flies exhibit significant increases in the size of the wings due to increased cell growth [11]. *Rps* have also been implicated as tumour suppressors in the vertebrate zebrafish model, where a genetic screen identified a link between malignant peripheral nerve sheath tumours and heterozygosity for several loss-of-function *Rp* mutations [12].

In mammalian systems, there is also evidence that *Rp* heterozygosity is frequently associated with tissue overgrowth and predisposition to cancer. For example, mutations in *RpS19*, *RpS17*, *RpS24*, *RpL35a*, *RpS7*, *RpL5*, *RpL11*, *RpS10* and *RpS26* have been associated with the human disease Diamond Blackfan Anemia (DBA), a dominant autosomal bone marrow failure syndrome, characterised by hypoplastic anemia with a predisposition to leukemia [13]–[19]. Mutations in *RpS14* are also associated with 5q- syndrome and predisposition to acute myeloid leukemia [20]–[21]. Although *RpS19* heterozygosity disrupts ribosome biogenesis 22–24, how reduced levels of Rps promote the excessive proliferation associated with progression to leukemia remains unclear and whether the mechanism is related to tissue overgrowth of *Minutes* has not been investigated.

Defining the mechanisms by which *Rp* heterozygosity results in tissue overgrowth and how reduction in a certain *Rp* gene predisposes a specific tissue to overproliferation in *Drosophila* is critical to understanding the processes linking growth and proliferation with tissue homeostasis. Furthermore, the insight provided by the *Drosophila* system may provide important clues in understanding how *Rp* mutations can promote cancer in humans.

Development of the *Drosophila* eye has been extensively used to identify and characterise regulators of growth and proliferation [25]–[26]. The *Drosophila* eye is composed of a highly organised array of photoreceptor clusters or ommatidia, which develop from an epithelial monolayer known as the eye imaginal disc. Differentiation of the ommatidia occurs in a wave that moves from the posterior toward the anterior. The anterior cells divide asynchronously and are separated from the differentiated posterior compartment by the morphogenetic furrow (MF) [27]. Mitotic division cycles become synchronized in the MF where cells are paused in G1 and a subset of photoreceptor cells are specified. The remaining retinal cells synchronously re-enter the cell cycle in the “Second Mitotic Wave” (SMW), which is composed of a tight band of DNA synthesis and mitosis. These final cell divisions provide the cells required for differentiation of the ommatidial structures that form the adult eye [28].

**Figure 1:**
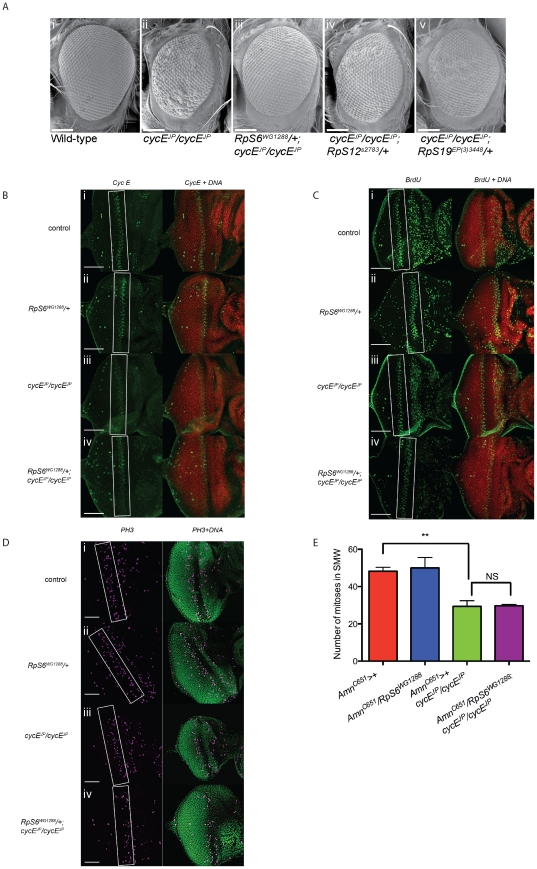
*RpS6* mutant suppresses the small rough eye phenotype of *cycE^JP^*, but not through restoring CycE protein levels. (A) Scanning electron micrographs (SEM) of female adult eyes with genotypes as indicated. Orientation of eyes: anterior (left) posterior (right). Scale bar 100 µm. (B) Confocal images of 3^rd^ instar eye imaginal discs stained for CycE and DNA with genotypes as indicated. White boxes mark the band of cycE cells. Images were taken at 40× magnification. Orientation of eye discs: anterior (left), posterior (right). Scale bar equals 50 µm. (C) Confocal images of 3^rd^ instar eye imaginal discs stained for BrdU incorporation and DNA with genotypes as indicated. White boxes mark the band of S phase cells. Images were taken at 40× magnification. Orientation of eye discs: anterior (left), posterior (right). Scale bar equals 50 µm. (D) Confocal images of 3^rd^ instar eye imaginal discs stained for cells in the SMW (PH3) and DNA with genotypes as indicated. White boxes mark the band of cells in SMW. Images were taken at 40× magnification with 0.7× optical zoom. Orientation of eye discs: anterior (left), posterior (right). Scale bar equals 50 µm. (E) Graph quantifying the number of cells in the SMW. Results are represented as the mean +/− standard error. Statistical analysis applied: unpaired t-test, where ** = p<0.01, NS = not significant and n = 3.

A hypomorphic mutation of *cycE*, *cycE^JP^* [29], reduces *cycE* expression during eye imaginal disc development to result in decreased S phases and small, rough adult eyes due to fewer cells (Figure 1A, compare i with ii) [29]. *cycE^JP^* therefore provides a sensitised genetic background to identify modifiers of eye proliferation, with suppressors of the phenotype being classed as “tumour suppressors” and predicted to normally function as cell cycle inhibitors [26]. To examine the mechanism(s) underlying the overgrowth phenotypes exhibited by some *Minutes* we have taken advantage of the unexpected observation that mutant *RpS6* suppresses the hypo-proliferative, small eye phenotype of *cycE^JP^* mutants [26]. The data presented here confirm that reduced function of *RpS6* suppresses the *cycE^JP^* small eye phenotype and we further demonstrate that this is not associated with restored proliferation in the SMW. Suppression of the *cycE^JP^* adult eye phenotype was observed with *Rp* mutants for both the small subunit (*RpS12* and *RpS19*) and the large subunit (*RpL38*), which suggests the ability to restore eye size may be a more general property of reduced Rp abundance. Further investigation revealed that reduced *RpS6* does not, however, lead to increased levels of CycE protein in the eye and that reduction of *RpS6* specifically in the eye does not suppress the *cycE^JP^* small eye phenotype. Instead we demonstrate that reduced *Rp* levels in the prothoracic gland in *RpS6* mutants decreased the activity of steroid hormone ecdysone, delayed development and hence allowed additional time for restoration of growth in the *cycE^JP^* mutants.

## Results

### *Rp* mutants suppress the *cycE^JP^* hypomorphic small eye phenotype

Mammalian *cyclin E* (*cycE*) is a well-characterised oncogene and, like the *Drosophila* homolog, regulates G1- to S-phase progression [30]–[32]. The *cycE^JP^* hypomorphic mutant has reduced *cycE* expression predominantly in the developing eye imaginal disc and, as a result, fewer S phases and small, rough adult eyes (Figure 1A ii and [29]). Previously a genetic screen for modifiers of the *cycE^JP^* phenotype identified the *RpS6* mutant *RpS6^air8^*, which reduces *RpS6* expression, as a suppressor of the *cycE^JP^* small eye phenotype [26]. This observation is consistent with previous observations that reduced *RpS6* expression can promote proliferation in *RpS6* mutant larvae [7]–[9].

We utilised the *cycE^JP^* small eye phenotype to examine the mechanisms by which reducing Rp levels can result in tissue overgrowth. As the original *RpS6^air8^* line was no longer available to confirm the previous findings [26], we demonstrated suppression of *cycE^JP^* using an alternate *RpS6* mutation, *RpS6^WG1288^* [8]–[9], which also exhibits the classic *Minute* phenotype of slender bristles (not shown) and a developmental delay (Figure 3C, red data points). *RpS6^WG1288^*/+ restored the eye size and reduced roughness in the *cycE^JP^* background to give adult eyes with a more wild-type appearance (Figure 1A, compare i and ii with iii). Thus, two independent *RpS6* mutations (*RpS6^air8^* and *RpS6^WG1288^*) suppress the *cycE* hypomorphic small eye phenotype, consistent with reduced *RpS6* function leading to increased proliferation in the *cycE^JP^* mutant.

**Figure 2:**
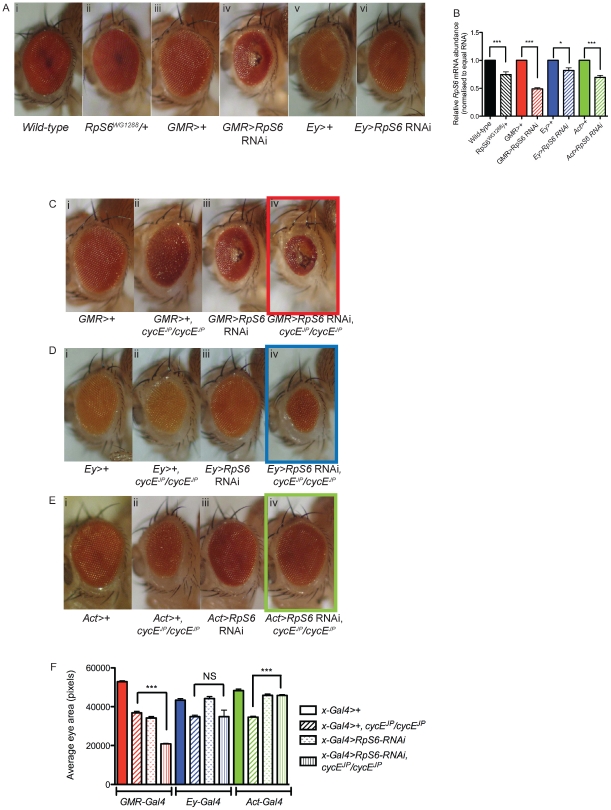
Reducing *RpS6* by RNAi in the whole fly, but not specifically in the eye, suppresses *cycE^JP^*. (A) Light micrographs of female adults bearing the genotypes as indicated. *GMR-Gal4* drives expression in differentiated eye photoreceptor cells. *Ey-Gal4* drives expression in all eye cells. (B) Graph showing the relative mRNA levels of *RpS6* from the *RpS6* mutant, eye specific reductions of *RpS6* (*GMR-Gal4* and *Ey-Gal4*) and ubiquitous reductions of *RpS6* (*Actin-Gal4*) as measured by qRT-PCR. RNA samples were extracted from ten 3^rd^ instar larvae or thirty 3^rd^ instar eye imaginal discs. Samples were normalised to equal amounts of RNA (1 µg). Results are represented as the mean +/− standard error (n = 3). Statistical analysis applied: One-way ANOVA, where * = p<0.05, *** = p<0.001. (C–E) Light micrographs of female adult eyes bearing the genotypes indicated. *Act-Gal4* drives expression in all cells. Orientation of eyes: anterior (left), posterior (right). (F) Graph of average eye area. (*GMR>+*) n = 13, (*GMR>+, cycE^JP^/cycE^JP^*) n = 13, (*GMR>RpS6* RNAi) n = 15, (*GMR>RpS6* RNAi; *cycE^JP^/cycE^JP^*) n = 19, (*Ey>+*) n = 21, (*Ey>+*; *cycE^JP^/cycE^JP^*) n = 11, (*Ey>RpS6* RNAi) n = 17, (*Ey>RpS6* RNAi; *cycE^JP^/cycE^JP^*) n = 12, (*Act>+*) n = 16, (*Act>+*; *cycE^JP^/cycE^JP^*) n = 42, (*Act>RpS6* RNAi) n = 31, (*Act>RpS6* RNAi; *cycE^JP^/cycE^JP^*) n = 49. Results are represented as the mean +/− standard error. Statistical analysis applied: One-way ANOVA, where *** = p<0.001 and NS = not significant.

In order to test whether suppression of *cycE^JP^* was specific to mutation of *RpS6* or was potentially a more general consequence of reducing *Rp* levels, we tested two other *Rp* mutants that give *Minute* phenotypes, *RpS12^s2783^* and *RpS19b^EP3448^*. Reducing *RpS12* and *RpS19* levels, with the mutant alleles *RpS12^s2783^* [33] and *RpS19b^EP3448^* (http://flybase.org/reports/FBrf0104946.html) resulted in a moderate suppression of *cycE^JP^* (Figure 1A iv and v, respectively). The *cycE^JP^* eye phenotype was also suppressed with a large subunit *Rp* mutant, *RpL38^2b1^* [11] (Figure S1). The finding that mutations in four different *Rps* from both subunits suppress the *cycE^JP^* phenotype suggests that this may be a common feature of *Minutes*.

### *RpS6* does not suppresses *cycE^JP^* by restoring Cyclin E protein levels in the eye

The majority of the suppressors examined in detail from the original *cycE^JP^* screen demonstrated the ability to restore CycE protein towards wild-type levels and an associated increase in S phase progression [26]. Thus we examined whether *RpS6^WG1288^* might similarly restore CycE levels in the eye. However, examination of CycE levels in eye discs from 3^rd^ instar larvae revealed that this was not the case (Figure 1B, compare i and iii with iv). As reported previously [29] and consistent with the reduced CycE levels, S phase cells were also reduced in eye discs of *cycE^JP^* (Figure 1C iii). In line with the finding that CycE was not altered, the reduced S phases in the SMW of *cycE^JP^* were not obviously increased by reducing *RpS6* (Figure 1C iv). Thus suppression of the *cycE^JP^* phenotype occurs in the absence of obvious changes to CycE abundance and S phase progression.

To monitor whether there was an overall change to cell cycle progression in the eye, we carried out anti-phosphohistone H3 staining to identify cells in mitosis as an alternative measure of cell cycles in the SMW (Figure 1D and quantified in 1E). The SMW of *cycE^JP^* mutants exhibited a significant reduction in their mitotic index as expected (Figure 1D iii and 1E). Importantly however the mitotic index was not restored in *cycE^JP^* eyes by the *RpS6* mutant (Figure 1D iv and 1E). Therefore in these animals there is not a significant increase in the rate of cell cycle progression in the SMW, which suggests that this is unlikely to be the mechanism underlying suppression of *cycE^JP^* by the *RpS6* mutant.

### Specific reduction of *RpS6* in the eye does not suppress *cycE^JP^*

The findings above suggested that the suppression of *cycE^JP^* by the *RpS6* mutant was not associated with either restoration of CycE or with altered cell cycle progression. As the *cycE^JP^* hypomorph predominantly affects the eye, we sought to test whether specific reduction of *RpS6* in the *cycE^JP^* eye could suppress the phenotype. Using the eye specific *GMR-Gal4* to drive expression of a *UAS-RpS6* RNAi transgene, in both the SMW and differentiated cells posterior to the morphogenetic furrow [34]–[35], resulted in a smaller eye with a glassy appearance and necrotic patches (Figure 2A, compare iii with iv) [36] and 50% reduction in *RpS6* mRNA in eye-antennal discs (Figure 2B). We then tested whether specific reduction of *RpS6* in the eye could suppress the *cycE^JP^* phenotype. Reducing *RpS6* with *GMR-Gal4*, which results in a small eye phenotype alone, was unable to suppress the *cycE^JP^* phenotype, and rather resulted in an additive reduction in eye size (Figure 2C, compare ii with iv). Due to the severity of the *GMR>RpS6* RNAi phenotype we also tested knockdown with an alternate eye driver *Ey-Gal4*, which is expressed in all eye cells [37]–[38]. This resulted in ∼20% reduction in *RpS6* mRNA in eye-antennal discs (Figure 2B) and did not produce an obvious adult eye phenotype alone (Figure 2A, compare v with vi). Thus like heterozygous *RpS6^WG1288^/+*, *Ey>RpS6* RNAi does not result in an obvious eye phenotype (Figure 2A, compare i with ii). However, in direct contrast to *RpS6^WG1288^/+, Ey>RpS6* RNAi enhanced rather than suppressed the *cycE^JP^* rough eye phenotype (Figure 2D, compare ii with iv). Together these data demonstrated that reducing the abundance of *RpS6* in the eye, either robustly or modestly, was unlikely to be the mechanism underlying suppression of the *cycE* hypomorphic phenotype by the *RpS6* mutant.

**Figure 3:**
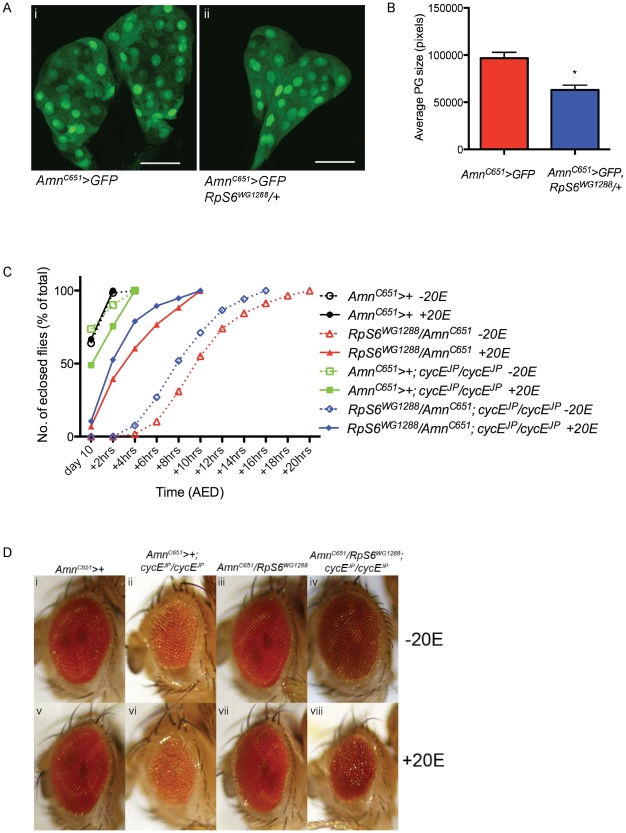
*RpS6* mutant larvae have smaller prothoracic glands and an ecdysone dependent developmental delay. (A) Confocal images of 3^rd^ instar prothoracic glands marked with GFP with genotypes indicated. Magnification 40×. Scale bar 50 µM. (B) Graph of average PG size. Results are represented as the mean +/− standard error. Statistical analysis applied: unpaired t-test, where * = p<0.05 (n = 3). (C) Graph representing the time to eclosion after egg deposition (AED) of genotypes indicated raised in the presence or absence of ecdysone (20E). *Amn^C651^-Gal4* drives expression in the prothoracic gland. (D) Light micrographs of female adult eyes bearing the genotypes indicated raised in the presence or absence of ecdysone (20E). Orientation of eyes: anterior (left), posterior (right).

### *RpS6* suppresses *cycE^JP^* in an eye tissue non-autonomous manner

Because specifically reducing *RpS6* in the eye did not suppress the *cycE^JP^* small eye phenotype, we considered the possibility that the interaction between *RpS6* and *cycE^JP^* might be mediated by a mechanism extrinsic to the eye. To test this we placed *UAS-RpS6* RNAi expression under the control of a range of ubiquitous *Gal4* drivers in an effort to replicate the environment of the *RpS6* mutant, by reducing *RpS6* in the whole fly. Knockdown of *RpS6* with the strong ubiquitous drivers *Daughterless-Gal4* or *Tubulin-Gal4* resulted in either early larval or embryonic lethality (Table S1). This is likely to be a result of *RpS6* levels dropping below the threshold required for sufficient ribosome assembly and thus protein synthesis to support cell growth and proliferation. Consistent with this observation, reduction of *RpS6* mRNA levels with strong drivers expressed in specific embryonic segments or larval domains also resulted in lethality (*Engrailed-Gal4*, *Patched-Gal4*) or shrivelled, stumpy wings (*MS1096-Gal4*) (Table S1 and Figure S2, compare iii with iv).

In contrast to the strong *Gal4* drivers, reducing *RpS6* mRNA levels with the relatively weaker ubiquitous driver, *Actin-Gal4*, resulted in viable flies (Figure S2, compare i with ii), which had a reduction in *RpS6* mRNA similar to the levels seen in *RpS6^WG1288^/+* larvae (Figure 2B, compare striped black and striped green bars). Importantly, this low-level reduction of *RpS6* throughout the fly resulted in suppression of the *cycE^JP^* eye phenotype (Figure 2E, compare ii with iv) and a significant increase in eye size (Figure 2F, green bars). These data suggested that factors extrinsic to the eye were essential for suppression of *cycE^JP^* by the *RpS6* mutant, consistent with our inability to detect changes in CycE activity or protein levels in the eye in the *RpS6* mutant background.

### Suppression of the *cycE^JP^* phenotype by the *RpS6* mutant is reversed by Ecdysone

As *Rp* mutations are associated with a developmental delay, we considered the possibility that the cell non-autonomous mechanism by which mutant *RpS6^WG1288^* and *RpS6* RNAi suppressed *cycE^JP^* might involve, at least in part, the ecdysone pathway, which is known to control timing of development and thus the growth period of the larvae. Specifically, release of ecdysone from the prothoracic gland (PG) dictates the timing of the metamorphosis from larvae to pupae (reviewed in [39]). As adult fly size is determined by the size of the larva at the time of pupal molt, the timing of ecdysone release plays a vital role in the growth of the fly [40]. We therefore examined whether *RpS6^WG1288^/+* might suppress the *cycE^JP^* eye phenotype via an ecdysone-dependent, cell non-autonomous mechanism.

Previous studies have reported a role for the PG as a size-assessment organ [41]–[43]. Inhibiting the growth of the PG causes an underestimation of body size and results in pupation at a larger size. Conversely, promoting the growth of the PG results in smaller flies [41]–[43]. For example, overexpression of a dominant negative isoform of *PI3* Kinase (*Dp110^DN^*) specifically in the PG blocks insulin pathway signalling and PG growth [41]. The smaller PG and associated reduction in ecdysone levels in these animals results in larger pupae and adults due to an extended larval growth period [41]–[43].

We therefore tested if the *RpS6* mutant might suppress the *cycE^JP^* phenotype by impairing PG growth and, as a consequence, affecting the level of ecdysone. During eye disc development the morphogenetic furrow moves forward by one row of ommatidia (3–4 cell rows) every 70 minutes [44] and the doubling time for cells in the proliferating, anterior portion of the eye disc is approximately 12 hours [45]. Thus a developmental delay would provide the anterior asynchronously dividing cells and the cells comprising the second mitotic wave of the eye imaginal disc extra time to grow and divide in order to compensate for the proliferation rate defect resulting from reduced CycE activity.

First, examination of heterozygous *RpS6* (*RpS6^WG1288^/+*) PGs, marked by expression of GFP, revealed that the glands were 35% smaller than GFP marked control PGs at the same time after egg deposition (AED) (Figure 3A, compare i with ii and quantified in 2B). This is also consistent with reports of *RpS6^air8^* mutant larvae having small, abnormal PGs [7]. As a direct consequence of reduced PG growth, it would also be expected that *RpS6^WG1288^/+* larvae should be developmentally delayed. Examination of developmental timing in *RpS6^WG1288^/+* heterozygotes revealed that reducing the levels of *RpS6* resulted in a delay in eclosion of up to 18 hours, compared to wild type (Figure 3C, compare open black circle with open red triangle). Importantly, the delay associated with the *RpS6* mutant is reduced by addition of the active form of ecdysone, 20-hydroxyecdysone (20E) (Figure 3C, red data points and statistical analysis shown in Table S3), which suggests the delay in the *RpS6* mutant is dependent on ecdysone levels.

The observation that the number of SMW divisions in the *RpS6^WG1288^*/+; *cycE^JP^/cycE^JP^* eyes were not significantly different to *cycE^JP^* alone suggests that the developmental delay and associated extra time for more cell divisions might underlie suppression of *cycE^JP^*. To investigate this possibility we tested whether suppression of *cycE^JP^* by the *RpS6* mutant was impaired when the developmental delay is reduced by addition of 20E (Figure 3D). First we demonstrated that the *RpS6^WG1288^*/+; *cycE^JP^/cycE^JP^* animals had a developmental delay comparable to that for the *RpS6* mutant alone, which could be reduced by the addition of ecdysone (Figure 3C, blue data points and statistical analysis shown in Table S3). Importantly, acceleration of development by the addition of 20E to the *RpS6^WG1288^*/+; *cycE^JP^/cycE^JP^* larvae resulted in a failure to suppress the small eye phenotype (Figure 3D, compare iv with viii). Thus suppression of the *cycE^JP^* phenotype by the *RpS6* mutant is dependent on a developmental delay, which is sensitive to the level of ecdysone.

### Reducing *RpS6* specifically in the prothoracic gland impairs growth and causes a developmental delay

**Figure 4:**
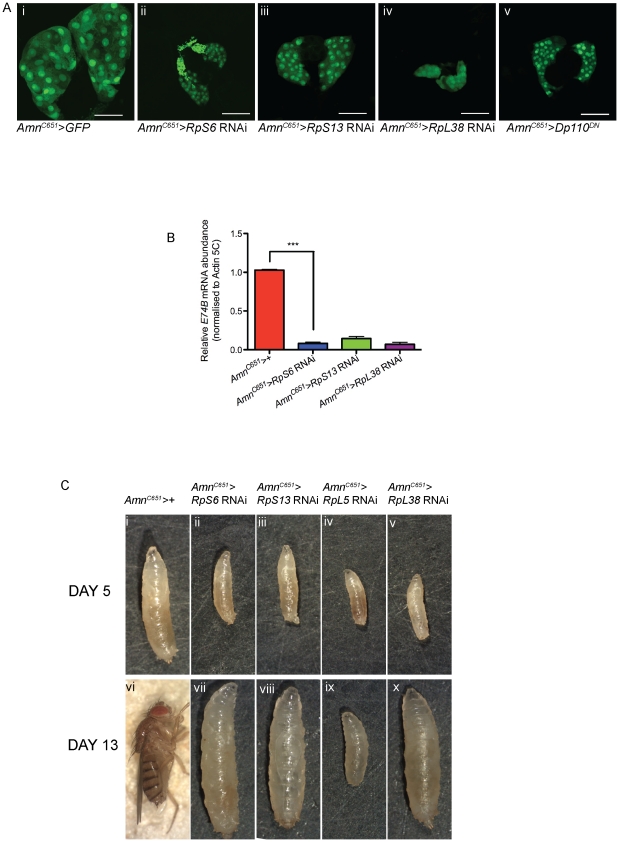
Reducing *Rps* by RNAi in the PG results in developmental delay and small prothoracic glands. (A) Confocal images of 3^rd^ instar prothoracic glands marked with GFP at day 5 with genotypes indicated. *Amn^C651^-Gal4* drives expression in the prothoracic gland. *Dp110^DN^* is a dominant-negative form of *PI3K*. Magnification 40×. Scale bar 50 µM. (B) qRT-PCR of relative mRNA levels of an ecdysone responsive gene *E74B*. RNA samples were extracted from 3^rd^ instar larvae. Samples were normalised to *Actin5C* mRNA levels. (*Amn^C651^>RpS6* RNAi) n = 4, (*Amn^C651^>RpS13* RNAi) n = 2, (*Amn^C651^>RpL38* RNAi) n = 2. Results are represented as the mean +/− standard error. Statistical analysis applied: unpaired t-test, where *** = p<0.001. (C) Light micrographs of 5 days AED larvae (i–v) or 13 days AED adult (vi) or delayed larvae (vii–x) with genotypes marked.

To further test our hypothesis that reduced levels of individual *Rps* in the PG of *Minute* mutants might restore proliferation in the *cycE^JP^* eye by inducing a developmental delay, we sought to reduce *Rp* expression in the PG using *Amn^C651^-Gal4* which drives expression in the PG [41] and *UAS-Rp* RNAi for *RpS6*, *RpS13* or *RpL38*. We first demonstrated the RNAi was able to reduce RpS6 protein by knocking down specifically in the PG, and staining with an anti-RpS6 antibody (Figure S3A). Consistent with the importance of Rps for growth, reducing *Rps* in the PG resulted in much smaller PGs in these larvae compared with the control at the equivalent time point of 5 days AED (Figure 4A ii–iv). Moreover, reduction of *RpS6* levels resulted in PGs that were smaller than for the *RpS6^WG1288^/+* PGs, suggesting a greater reduction in *RpS6* (compare Figure 4A ii to Figure 3A ii). Examination of the *Amn^C651^>RpS6* RNAi PGs at 12 days AED revealed that the size of the gland was still considerably smaller than the control PG (data not shown). As a smaller PG would be predicted to result in less ecdysone synthesis and release, we examined if the reduction in PG size affected ecdysone activity in the larvae. qRT-PCR was performed on whole larvae to measure ecdysone activity indirectly by quantifying the mRNA levels of an ecdysone responsive gene, *E74B* [41]. *E74B* levels were normalised to *Actin-5C*, a non-ecdysone responsive gene. RNAi-mediated reduction of *RpS6*, *RpS13* or *RpL38* in the PG resulted in up to 90% decrease in *E74B* expression (Figure 4B), suggesting strongly reduced ecdysone activity, reflecting the small size of the PG.

Consistent with the robust reduction in PG size and reduced ecdysone activity, we observed an extreme developmental delay in the larvae with RNAi-mediated knockdown of *RpS6*, *RpS13* or *RpL38* in the PG. At day 5, these larvae were smaller in size compared with control larvae (Figure 4C, compare i with ii–v). While the control larvae underwent pupation as normal at day 5, larvae with reduction of *RpS6*, *RpS13* or *RpL38* specifically in the PG continued to feed and grow beyond day 10 to become giant larvae, which fail to pupate (Figure 4C, compare vi with vii–viii, x). The phenotype for the *RpL5* knockdown in the PG was even more dramatic, being 2^nd^ instar larval lethal (Figure 4C ix), suggesting that *RpL5* was knocked down below the threshold required for cell intrinsic growth [36], [46]–[47] and, therefore, development of the PG gland. This is consistent with the lethality that results when strong drivers are used to express RNAi transgenes targeting the Rps investigated here (Table S2).

The *Amn^C561^-Gal4* insertion is not expressed solely in the PG, being expressed throughout the ring gland early, in some cells in the ventral ganglion and in neurosecretory cells of the brain [41]. As the neurosecretory cells of the brain can also play a role in developmental timing and growth [48], we addressed the possibility that *RpS6* knockdown in these cells might be responsible for the overgrowth by using another driver, *P0206-Gal4* [43], that also expresses in the PG, but not in the neurosecretory cells. Consistent with the effect being mediated through defects in PG development, knockdown of either *RpS6* or *RpL38* using *P0206-Gal4* also resulted in an extreme developmental delay whereby larvae continue to feed for greater than 20 days and fail to pupate, which was associated with a smaller PG (Figure S4A).

### The impaired growth and developmental delay is mediated by ecdysone

**Figure 5:**
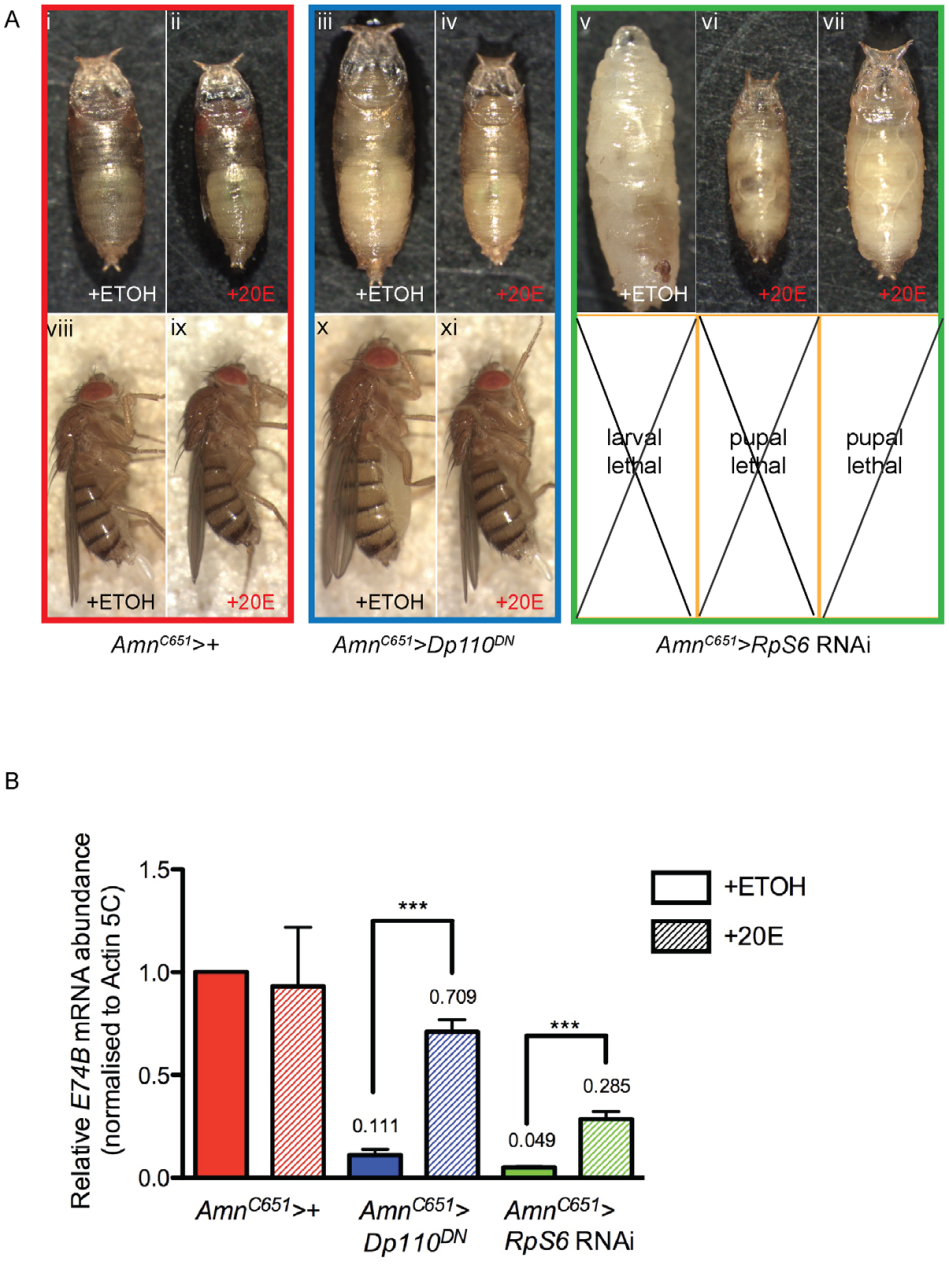
Addition of 20-hydroxyecdysone can partially rescue *Amn>RpS6* RNAi larval lethality. (A) Light micrographs of day 8 pupae/larvae or day 15 female adults bearing the genotypes indicated. The larvae were fed 0.75 mg/mL of 20E or equivalent concentration of 7.5% (v/v) ETOH. (B) qRT-PCR of relative mRNA levels of an ecdysone responsive gene *E74B* from larvae with or without 0.75 mg/mL of 20E. RNA samples were extracted from 3^rd^ instar larvae. Samples were normalised to *Actin5C* mRNA levels. Results are represented as the mean +/− standard error (n = 3). Statistical analysis applied: unpaired t-test, where *** = p<0.001.

To assess whether the reduced ecdysone production was the cause of the developmental delay and larval overgrowth resulting from *Rp* knockdown in the PG, 20E was introduced to the food of *Amn^C651^>RpS6* RNAi larvae (Figure 5A). The addition of 20E resulted in a variable restoration of pupariation, which ranged from progression towards cuticle darkening in larvae to cuticle development and early pupal morphology (Figure 5A, compare v with vi–vii and Figure 5B green bars). Although the *Amn^C651^>RpS6* RNAi larvae were able to pupate, the ectopic addition of 20E was unable to initiate the final steps of metamorphosis, including the formation of adult structures. This suggests that ∼30% of the endogenous 20E activity achieved by feeding the larvae (Figure 5B) is sufficient to trigger pupariation, but is below the threshold required for adult metamorphosis. The failure of metamorphosis may be confounded by the fact that pupae, unlike larvae, can no longer take up 20E by feeding. Indeed, the largest peak of endogenous ecdysone release occurs after cuticle formation and is required for the formation of adult structures [39].

To confirm this failure to restore pupation was not due to insufficient 20E in the food we carried out a control rescue experiment with an alternate growth regulator, PI3K, which has previously been shown to modulate PG size and development [41]. Despite having a PG size similar to that of *Amn^C651^>RpS6* RNAi (Figure 4A, compare ii with v) and associated extreme developmental delay, the *Amn^C651^>Dp110^DN^* (dominant negative PI3K) larvae were only moderately delayed and pupated, but eclosed as larger flies (Figure 5A, compare viii with x, and [41]). We demonstrated that feeding 20E to larvae overexpressing dominant negative *PI3K* in the PG (*Amn^C651^>Dp110^DN^*) restored the time of pupation back to day 5, the adults eclosed at a normal size (Figure 5A, compare iii and x with iv and xi), and *E74B* levels were significantly increased compared to that of control (Figure 5B, blue bars). This restoration of timing and size toward control suggested that the 20E was successfully taken up and processed by the *Amn^C651^>Dp110^DN^* larvae. The difference in the severity of the phenotypes in terms of developmental delay, strongly suggested that ecdysone levels are more sensitive to disruption of *Rps* and ribosome biogenesis than to disruption of insulin pathway-dependent growth in the PG

### Reducing *RpS6* levels using RNAi in the prothoracic gland in *cycE^JP^* background suppresses the *cycE^JP^* phenotype

**Figure 6:**
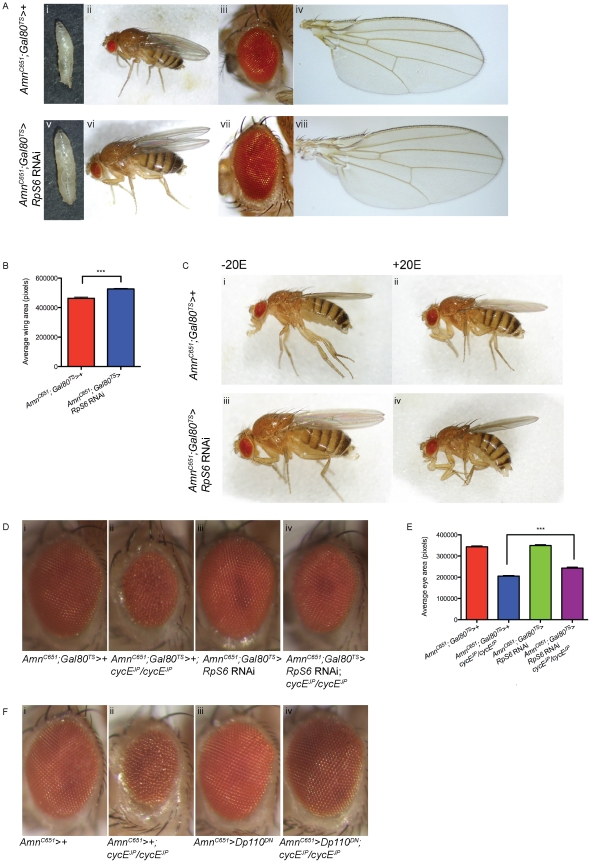
Reducing *RpS6* in the PG is associated with tissue overgrowth and suppresses *cycE^JP^*. (A) Light micrographs of larvae, whole adult flies, adult eyes and wings bearing the genotypes: (i–iv) control (*Amn^C651^; Tubulin-Gal80^TS^>+*) and (v–viii) delaying reduction of *RpS6* in the PG until 2^nd^ instar (*Amn^C651^; Tubulin-Gal80^TS^>RpS6* RNAi). (B) Graph of average wing area. (*Amn^C651^; Tubulin-Gal80^TS^>+*) n = 7, (*Amn^C651^; Tubulin-Gal80^TS^>RpS6* RNAi) n = 10. Results are represented as the mean +/− standard error. Statistical analysis applied: unpaired t-test, where *** = p<0.001. (C) Light micrographs of female adult flies bearing the genotypes indicated raised in the presence or absence of ecdysone (20E). (D) Light micrographs of female adult eyes bearing the genotypes indicated. Orientation of eyes: anterior (left), posterior (right). (E) Graph of average eye area. (*Amn^C651^; Tubulin-Gal80^TS^>+*) n = 26, (*Amn^C651^; Tubulin-Gal80^TS^>+; cycE^JP^/cycE^JP^*) n = 26, (*Amn^C651^; Tubulin-Gal80^TS^>RpS6* RNAi) n = 31, (*Amn^C651^; Tubulin-Gal80^TS^>RpS6* RNAi; *cycE^JP^/cycE^JP^*) n = 19. Results are represented as the mean +/− standard error. Statistical analysis applied: unpaired t-test, where *** = p<0.001. (F) Light micrographs of female adult eyes bearing the genotypes indicated. Orientation of eyes: anterior (left), posterior (right).

As *RpS6* knockdown in the PG gland resulted in a failure to undergo pupation, in order to carry out further studies we examined whether we could reduce the severity of the phenotype and facilitate development into adult stages using a temperature sensitive isoform of the *Gal4* repressor, *Gal80* (*Gal80^TS^* [49]) that allows temporal control of the induction of *RpS6* knockdown by RNAi in the PG. Thus, knockdown of *RpS6* was delayed until late 2^nd^ instar and although this still resulted in large, developmentally delayed larvae (Figure 6A, compare i with v), these larvae were able to undergo pupation and eclosed as large adults (Figure 6A, compare ii with vi). In addition, we observed increases in the eye size (Figure 6A, compare iii to vii) and statistically significant increase in the wing size (Figure 6A, compare iv to viii, quantified in 6B), in the *Amn^C651^;Gal80^TS^>RpS6* RNAi adults compared with control.

We then tested whether we were able to alter this overgrowth by the addition of ecdysone. Indeed, addition of 20E to the *Amn^C651^;Gal80^TS^>RpS6* RNAi restores the adults to a similar size to the *Amn^C651^;Gal80^TS^* control animals (Figure 6C, compare ii to iv). This suggests that the overgrowth also depends on reduced levels of ecdysone activity, as observed for the *Amn^C651^>Dp110^DN^* animals (shown in Figure 5A xi where body size is similar to control in *Amn^C651^>Dp110^DN^* +20E, Figure 5A ix). Thus the overgrowth phenotype resulting from reduction of *RpS6* in the PG was sensitive to the level of 20E, which supports the hypothesis that the developmental delay associated with knockdown of *RpS6* specifically in the PG is due to impaired ecdysone release and delayed metamorphosis.

Most importantly, reduction of *RpS6* in the PG resulted in suppression of the *cycE^JP^* eye phenotype, with a statistically significant increase in adult eye size (Figure 6D, compare ii with iv, and quantified in 6E). Thus, the ability of the *RpS6* mutant to suppress the *cycE^JP^* phenotype occurs, at least in part, through a defect in PG growth and the associated delay in development. The suppression by PG-driven *RpS6* knockdown was not as strong as observed for the *RpS6* mutant, which could be a consequence of the severe reduction in 20E activity in these animals (Figure 4B). As ecdysone release is required for proper morphogenetic furrow progression in eye discs [50], the drastic reduction in 20E levels in the PG-driven *RpS6* RNAi animals, specifically in a background of diminished CycE levels, might also delay furrow progression. Thus, even though extra time is spent during the larval growth period, the suppression is incomplete because of the role of 20E in controlling the developmental signals required for furrow progression [50]–[51].

**Figure 7:**
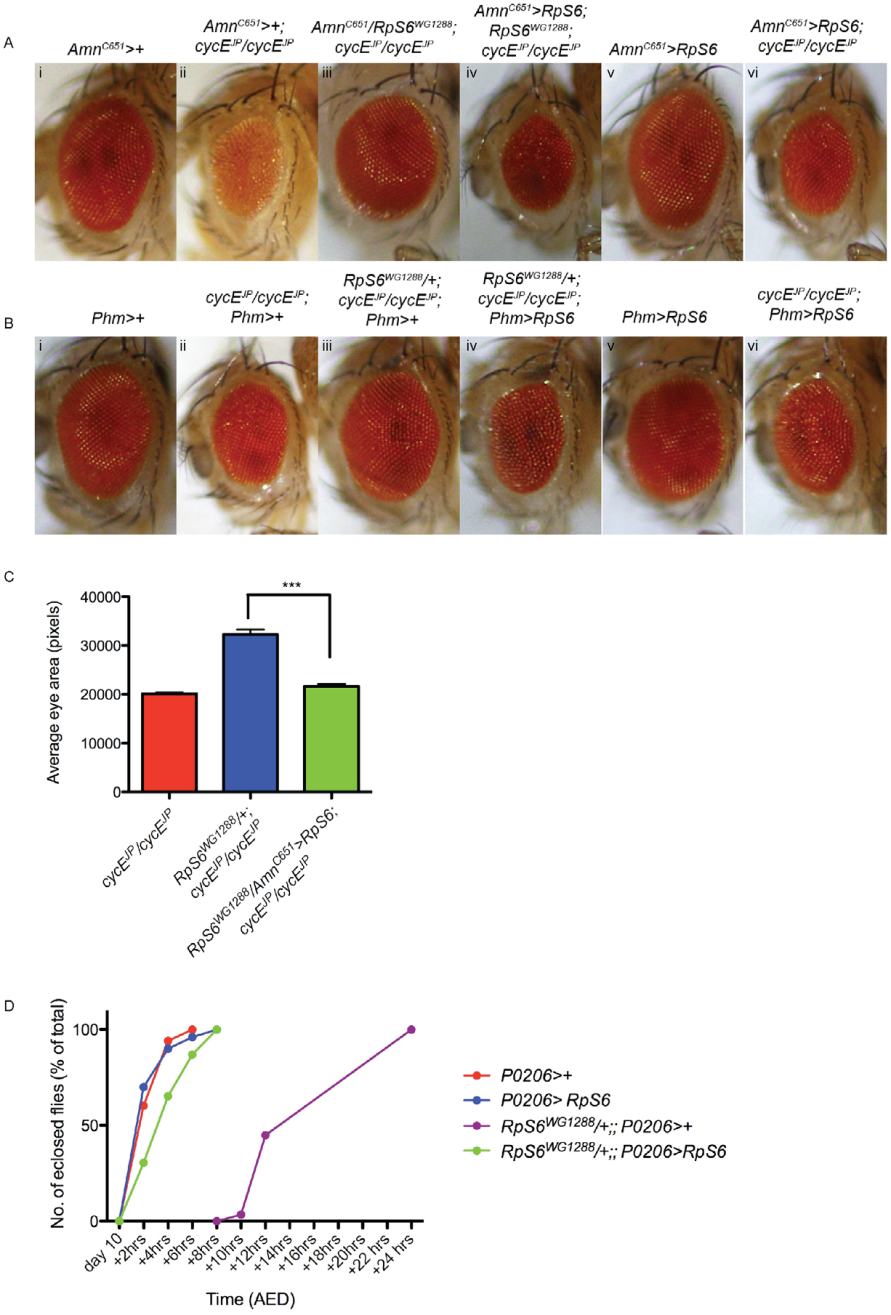
Restoring *RpS6* expression in the PG inhibits the suppression of *cycE^JP^* by the *RpS6^WG1288^* mutant. (A,B) Light micrographs of female adult eyes bearing the genotypes indicated. Orientation of eyes: anterior (left), posterior (right). *Amn^C651^-Gal4* and *Phm-Gal4* drive expression in the prothoracic gland. (C) Graph of average eye area. (*Amn^C651^>+; cycE^JP^/cycE^JP^*) n = 7, (*RpS6^WG1288^/+; cycE^JP^/cycE^JP^; Phm>+*) n = 6, (*RpS6^WG1288^/Amn^C651^>RpS6; cycE^JP^/cycE^JP^*) n = 14. Results are represented as the mean +/− standard error. Statistical analysis applied: unpaired t-test, where *** = p<0.001. (D) Graph representing the time to eclosion after egg deposition (AED) of genotypes indicated. *P0206-Gal4* is a ring gland specific driver.

These data strongly support a model whereby *RpS6^WG1288^/+* suppresses the small rough eye phenotype of *cycE^JP^* via a cell non-autonomous mechanism. Reduced abundance of *RpS6* in the PG of *cycE^JP^* animals decreases PG size, ecdysone activity and consequently results in a developmental delay and time for additional growth of the eye. To definitively test this model, we examined the effect of restoring *RpS6* expression in the PG of *RpS6^WG1288^/+*; *cycE^JP^*/*cycE^JP^* flies. According to the model above, if the decrease in *RpS6* expression specifically in the PG is responsible for the ability of *RpS6^WG1288^/+* to suppress the small *cycE^JP^* eye phenotype, then we would predict that restoring *RpS6* expression specifically in the PG in the *RpS6^WG1288^/+*; *cycE^JP^/cycE^JP^* flies would prevent the developmental delay and inturn prevent the suppression of the small eye phenotype. Consistent with this, expression of *RpS6* using the *Phantom-Gal4* (*Phm-Gal4*) driver [43], a PG specific driver, resulted in ectopic expression of *RpS6* in the PG (Figure S3B). Similar results were shown for enforced expression of *RpS6* in the PG using PG driver *Amn^C651^-Gal4*. Restoration of expression of *RpS6* in the PGs of *RpS6^WG1288^/+*; *cycE^JP^/cycE^JP^* flies using either the *Amn^C651^-Gal4* (Figure 7A, compare iii with iv) or *Phm-Gal4* driver (Figure 7B, compare iii with iv) prevented *RpS6^WG1288^* from suppressing the *cycE^JP^* eye phenotype (quantified in Figure 7C). Subsequent studies demonstrated this was because enforced expression of *RpS6* in the PG's of *RpS6^WG1288^* animal prevented the developmental delay (Figure 7D, green data points and statistical analysis shown in Table S4). Together these data are consistent with the above model and unequivocally demonstrate that the ability of the *RpS6^WG1288^/+* mutant to suppress the *cycE^JP^* phenotype is due to reduction of *RpS6* abundance specifically in the PG.

In summary, these data strongly support the hypothesis that the ability of the *RpS6* mutant to suppress the *cycE^JP^* small rough eye phenotype is, in large part, due to a reduction of PG size and an associated decrease in ecdysone activity, which results in an extended larval growth period that allows the eye discs extra time to grow. This model predicts that manipulation of other growth modulatory genes in the PG would also suppress the *cycE^JP^* phenotype. Indeed, consistent with this model, overexpression of *UAS-Dp110^DN^* in the PG was also able to suppress the *cycE^JP^* small rough eye phenotype (Figure 6F, compare ii with iv). As observed for the *RpS6* mutant, CycE protein levels, BrdU and PH3 in the *Amn^C651^>Dp110^DN^*, *cycE^JP^/cycE^JP^* eye imaginal discs were not altered compared with *cycE^JP^* alone (Figure S5). As we do not see a significant increase in the SMW divisions in these animals, when compared with *cycE^JP^* alone, this further supports the idea that the increased time spent in the larval growth stage allows more time for division, which leads to suppression of the small eye phenotype.

## Discussion

Since the *Minutes* were first described in 1929 [2], geneticists have sought to understand the mechanisms underlying these phenotypes as an avenue toward elucidating the complex mechanisms controlling body size. More recently, heterozygous mutations in multiple *Rp* genes have been associated with overgrowth phenotypes [11]–[12], [20], but the underlying mechanism has remained poorly understood. We addressed this question here taking advantage of a genetic screen for modifiers of a *cycE* hypomorph, which identified an *RpS6* mutant as a suppressor [26], to investigate possible mechanisms by which *Rp* mutations may contribute to overgrowth.

### The cell non-autonomous model for suppression of *cycE^JP^* and overgrowth phenotypes in *Minutes*

**Figure 8:**
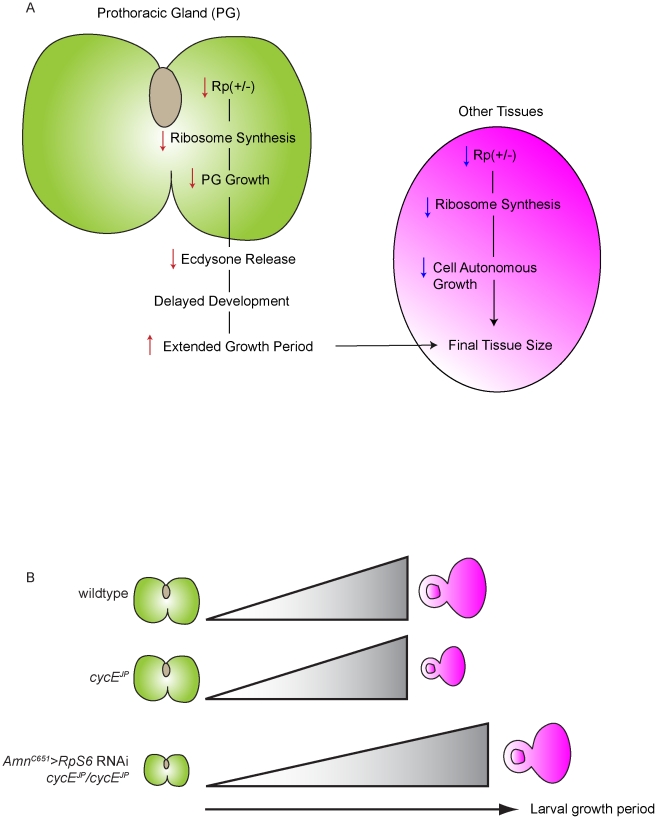
The ecdysone model of *cycE^JP^* suppression and *Minute* overgrowth phenotype. (A) Diagram of the two effects of *Rp* reductions in *Drosophila*. First is the intrinsic effect of reducing *Rps* in the prothoracic gland (PG). The second is an extrinsic effect on the target tissue. The final size of the adult fly is the net consequence of both effects. (B) Model for suppression of *cycE^JP^* via altered PG size and ecdysone activity. In wild-type PGs, ecdysone titres accumulate and allow normal growth of the eye imaginal disc (depicted by the grey gradient). In *cycE^JP^* eye discs, while the PG size is normal, the eye discs have reduced proliferation/growth due to the *cycE^JP^* mutation. Reduction of *RpS6* reduces PG size and ecdysone activity to cause an extended larval growth period, allowing extra time for the *cycE^JP^* eye discs to grow.

Our data demonstrate that *Rp* mutants suppress the *cycE* phenotype via a mechanism extrinsic to the eye, involving control of developmental timing though the PG. We propose the following model to explain this phenomenon (Figure 8). Firstly, reduced *Rp* levels in the PG of *Rp* mutant flies decreases ribosome biogenesis thus inhibiting PG growth, which in turn results in reduced ecdysone synthesis and a subsequent delay in development (Figure 8A). The extended growth period resulting from the developmental delay allows time for more cell divisions and growth in the eye, thereby allowing the eye imaginal disc to achieve normal size prior to pupation, thus suppressing the *cycE^JP^* small eye phenotype (Figure 8B). In support of the tissue extrinsic component of PG-ecdysone model, we have demonstrated that reducing *RpS6* specifically in the PG suppresses *cycE^JP^* (Figure 6D), and conversely overexpression of *RpS6* in the PG prevents suppression of the *cycE^JP^* by mutant *RpS6* (Figure 7A–7B).

As a developmental delay is a consistent feature of *Minutes*, it was speculated by Brehme in 1939 that this aspect of the phenotype might be due to insufficient ecdysone (as reviewed in [47]). Our work confirms this hypothesis and importantly, also provides a framework for how the Rp *Minute* collection of mutants are associated with both impaired growth and, counter-intuitively, tissue overgrowth (Figure 8A). In essence final tissue/body size in a *Minute* fly is a product of interplay between the tissue intrinsic effect of altering Rp levels in the cells of individual tissues and the extrinsic effects of *Rp* mutants on hormone release (Figure 8A) and thus developmental timing. As Rps and the rRNAs are required in equimolar amounts to form functional ribosomes, the relative contribution of tissue intrinsic versus extrinsic growth requirements to final tissue/body size would be dependent on the expression level and stability of each *Rp*, which will dictate whether levels of the specific Rp are rate-limiting for ribosome biogenesis in a given tissue. Enlargement of tissues for any given *Minute* would only occur if reduction of the Rp in the affected tissue did not reduce levels below those required for tissue growth. If Rp levels were below the threshold in a particular tissue, its growth would be inhibited, effectively negating the effects of an increased larval growth period provided by the developmental delay. This is consistent with the observation that expression of a given *Rp* mRNA varies between tissues [52]–[54], indicating that a particular Rp may be rate limiting for proliferative growth in one tissue but not in another. For example, while all of the *Minutes* are developmentally delayed, wing overgrowth has not been widely described, suggesting that the reduced levels of the relevant Rp associated with the *Minute* in question are limiting in both the wing and PG. In contrast, *RpL38^2b1^/+* and *RpL5^2d2^/+* flies have overgrown wings [11] which suggests that the reduced level of RpL38 associated with *RpL38^2b1^/+* flies is not limiting for proliferative growth in wing discs but is limiting for PG growth, thus the extended growth period results in larger adult wings. Therefore the final size of the *Minute* and its individual tissues is the net effect of both the tissue extrinsic effects of reducing *Rps* in the PG, and the tissue intrinsic effects of reducing *Rps* in the cells of other tissues (Figure 8A).

The mechanisms behind maintaining body/organ size are complex, and in addition to intrinsic cellular growth rate and the time spent in the growth phase prior to pupation described above, recent studies of imaginal disc regeneration reveal that the final size of *Drosophila* imaginal tissues is sensitive to an overarching mechanism that slows the division rate of the non-regenerating compartments even in the event of developmental delay [55]. This may explain why the *RpS6^WG1288^/+* mutant is able restore eye size back toward the wild type size in a background sensitised to impaired eye growth, ie., the *cycE^JP^* background, but does not normally lead to eye overgrowth or overgrowth of other tissue compartments, despite being associated with a developmental delay.

Clearly however, these final size constraints can be overridden or are not triggered in certain *Minutes* eg., *RpL38^2b1^/+* and *RpL5^2d2^/+* flies which have overgrown wings. In these cases the ongoing wing imaginal disc growth occurring during the extended larval period appears to be sufficient to overcome the normal size control checkpoints that normally restrict overgrowth of this tissue. Consistent with this model, knockdown of *RpS6* or *RpL38* specifically in the PG rather than the whole fly using the ring gland driver (*P0206-Gal4*) results in a smaller PG and developmental delay, which is associated with overgrown larvae (Figure S4A) and for *RpL38* with significantly increased wing imaginal disc size (Figure S4B–S4C).

Together these findings demonstrate the complexities of the cell non-autonomous effects of *Rp* reduction on tissue growth, which has implications for many of the experimental manipulations carried out by *Drosophila* researchers. For example if mosaic clones are generated in the whole animal using the *Minute* technique to maximize size of mosaic clonal tissue, this might also impact on PG growth and have unforeseen cell non-autonomous effects on the tissue of interest, which will need to be taken into consideration.

### The relationship between overgrowth in *Minutes* and predisposition to cancer associated with *Rp* haploinsufficiency in vertebrates

Our studies also raise the interesting question of whether the cell non-autonomous mechanisms underlying tissue overgrowth phenotypes of *Minutes* described here are relevant to the mechanisms responsible for tissue-specific phenotypes associated with *Rp* mutations in vertebrates. These ribosomopathies [56] include abnormal erythrocyte maturation, thrombocytosis and a predisposition to leukemia, associated with *Rp* haploinsufficiency syndromes such as the 5q- syndrome and Diamond-Blackfan anaemia (DBA) in humans [13], [20] or nerve sheath tumours in fish [12]. We think the cell non-autonomous mechanism described herein is unlikely at least for the 5q- syndrome, as the pathogenesis of ribosomal protein-mediated bone marrow failure appears to be largely cell intrinsic involving ribosomal stress mediated activation of p53 and defective development of haematopoietic system [57]. This is not to say that cell extrinsic effects of ribosomopathies may not contribute to development defects and disease at some level in vertebrates, for example, through defective growth of tissues important for release of paracrine or endocrine acting hormones. Clearly additional studies are required to determine to what extent altered *Rp* gene dosage contributes to human disease other than bone marrow failure and whether they are mediated by cell intrinsic or extrinsic mechanism or, indeed both.

In summary, our findings establish that suppression of *cycE^JP^* by the *RpS6* mutant is exerted via a mechanism wherein reduced *Rp* levels in the prothoracic gland decreases abundance of the steroid hormone ecdysone, delaying development and hence allowing additional time for tissue and organ overgrowth. These data provide for the first time a rationale to explain the counter-intuitive organ overgrowth phenotypes observed for certain *Drosophila Rp* mutants. Furthermore, they provide new insight into mechanisms governing tissue size homeostasis, suggesting that different tissues may exhibit distinct thresholds of expression of individual *Rps*. Thus, regulated expression of individual *Rps* could exert tissue specific effects on cell growth and organ size.

## Materials and Methods

### *Drosophila* stocks and culture

Unless otherwise stated the fly strains used were obtained from the Bloomington Stock Center and are described in FlyBase (http://flybase.org). The *UAS-RpS6* transgenic lines for overexpression were generated by cloning the full-length *RpS6* cDNA into pUAST and then injected into *Drosophila* embryos, as previously described in [58]. The following strains were described in: *w^−^;cycE^JP^* [29], *Amn^C651^-Gal4* [41], *Phm-Gal4* [43], *P0206-Gal4* [42]–[43], *UAS-Dp110^DN^* [59], *UAS-Ras^V12^* [60], *UAS-Cyclin E* [61], *UAS-p35* [62], *GMR-p21* [63], *UAS-cycD* and *UAS-cdk4* [64].

### Generation of ribosomal protein RNAi transgenic flies

*RpS6* RNAi construct: the longest open reading frame for *RpS6* (654 bp) was PCR amplified with primers 5′-CTGCAGGAATTCGGACAGGTTGTGGAGGCCGAT-3′ and 5′-GGTACCGAATTCTTACTTCTTGTCGCTGGAGACAG-3′ (*EcoRI* sequence underlined) and PCR products were digested with *EcoRI* and ligated into the SYMpUAST vector [65].

*RpS13, RpL5, RpL30 and RpL38* RNAi constructs: products were digsted with *XbaI* and inserted into pWIZ as inverted repeats in a two–step cloning process [66]. *RpS13*: the 302 bp coding region of the 3^rd^ exon was PCR amplified with primers 5′-ATATTCTAGAGCATCATCCTGCGTGACTCGC-3′ and 5′-ATATTCTAGAGGCAACCAGGGCGGAGGC-3′ (*XbaI* sequence underlined). *RpL5*: the 264 bp coding region of the 2^nd^ exon PCR amplified with primers 5′-GCGCTCTAGAGGTTTCGTTAAGGTAGTC-3′ and 5′-GCATTCTAGACTGGATCCCGTATTTGGG-3′. *RpL30*: the 199 bp 5′UTR and coding region of the 1^st^ exon was PCR amplified with primers 5′-GCATTCTAGATCGCCTGCAGTGCTTTAACC-3′and 5′-ATATTCTAGACTCAGGGCGGGCGTGTTGC-3′. *RpL38*: the 213 bp coding region of the 2^nd^ exon was PCR amplified with primers 5′-GCGCTCTAGAATGCCACGGGAAATTAAAG-3′ and 5′-GCGCTCTAGATTATTTCACCTCCTTTAC-3′. All constructs were injected into *Drosophila* embryos, as previously described in [58].

### Temperature shift experiments with *Gal80^TS^*

Conditional expression of *UAS-RpS6* RNAi was carried out using a temperature sensitive isoform of *Gal80*, the repressor of *Gal4* (*Gal80^TS^* [49]). Larvae were raised at the permissive temperature of 18°C and shifted at late 2^nd^ instar to the restrictive temperature of 25°C.

### Assessing developmental delay

For each experiment, forty 1^st^ instar larvae were collected 24 hour AED (0–4 hour collections) from lay cages with grape agar plates. To measure time of eclosion, vials were checked for the number of eclosed adults every 2 hours from 10 days AED until adult flies no longer emerged. For 20-hydroxyecdysone treatment twenty 1^st^ instar larvae were collected 24 hour AED (0–4 hour collections) and transferred into vials containing yeast paste supplemented daily with 0.75 mg/ml of 20-hydroxyecdsyone (Sigma).

### Microscopy and imaging

Antibody staining, BrdU labelling and quantification were carried out as described previously [67]–[68]. Antibodies used were the anti-RpS6 polyclonal (raised in mice), anti-bromodeoxyuridine (Becton Dickinson), PH3 (Upstate) and anti-cycE (rat) (a gift from Helena Richardson). Serial sections of eye imaginal discs or prothoracic glands were taken on a Zeiss Imager Z1 using the LSM 510 Meta software. Image preparation and analysis were conducted in Adobe Photoshop CS2 v9.0 and ImageJ v1.37.

For light microscopy images were captured on an Olympus DP11 camera. Female adult eyes were imaged at 5.6× magnification, and larvae or adult flies were imaged at 1.6× magnification. All images were processed using Adobe Photoshop. Eye area was measured by tracing around the perimeter of the photoreceptor cells of cropped images using Metamorph Offline version 7.6.3.0 software.

For electron microscopy female adult flies were progressively fixed in 25% (v/v) acetone for 1 hour nutation at room temperature, 50% (v/v) acetone for 1 hour nutation at room temperature, 75% (v/v) acetone for 1 hour nutation at room temperature, and finally stored in 100% acetone. The sample was then critical point dried on a Balters CPD 030 Critical Point Dryer and coated with gold particles in an Edwards 6150B Gold Sputter Coater. Images were recorded on a Phillips XL30 FEG Field Emission Electron Microscope.

### Prothoracic gland size measurements

For measurements of prothoracic gland (PG) area size, confocal images of PGs taken at 40× magnification were quantified with BB Thermometer v1.1 Software (BenBritten.com).

### Wing size measurements

Adult wings were mounted into Canada Balsam and xylene. Images were taken at 4.5× magnification. Whole wing area was measured using the magnetic lasso tool and record measurement function of Adobe Photoshop.

### Reverse Transcriptase–PCR (RT–PCR)

Total RNA was isolated from ten 3^rd^ instar larvae or thirty 3^rd^ instar eye imaginal discs with TRIzol (GibcoBRL) following manufacturer's instructions. cDNA was synthesised from 1 µg RNA using the Superscript First Strand synthesis system for RT-PCR (Invitrogen) following the manufacturer's guidelines. qRT-PCR was carried out with SYBR Green under standard conditions in the Step One Plus Real Time PCR system (Applied Biosystems)

Primer sequences were as follows:

*RpS6* forward (TGTTCCAGCTCAACGTTTCCT)

*RpS6* reverse (TCGTCGACCACTTCGAATAGC)

*Actin 5C* forward (CCCCAAGGCCAACCGTGAGA)

*Actin 5C* reverse (ACCCGAAGCGTACAGCGAGAGC)

*E74B* primers as published in [41].

Amplicon specificity was verified by melting curve analysis (65 to 90°C) after 40 cycles. An average Ct value for the three technical replicates was calculated for each sample. The fold change expression of *RpS6* mRNA levels was normalised to equal RNA and determined using the 2^−ΔΔCT^ method. *E74B* mRNA levels were normalised to *Actin 5C* mRNA levels of untreated control cells and determined using the 2^−ΔΔCT^ method [69].

### Statistical analysis

Statistical analysis was performed in GraphPad Prism software using either Unpaired t-test or One-way ANOVA, with Tukey's test for multiple comparisons, as stated in figure legends.

## Supporting Information

Figure S1 *RpL38^2b1^* suppresses *cycE^JP^*. Light micrographs of female adults bearing the genotypes indicated.
(TIF)

Figure S2 Reducing *RpS6* in different tissues by RNAi. Light micrographs of female adults bearing the genotypes indicated.
(TIF)

Figure S3 RpS6 protein is knocked down by *UAS-RpS6* RNAi and overexpressed by *UAS-RpS6*. (A,B) Confocal images of 3^rd^ instar prothoracic glands at day 5 stained for anti-RpS6 antibody and DNA, genotypes marked. *P0206-Gal4* is a ring gland specific driver [42]–[43]. *Phm-Gal4* is a PG specific driver [43]. Confocal images were taken at equivalent settings (Zeiss Meta settings, pinhole 1.2, gain 525) for comparison between the *UAS-RpS6* RNAi and control. Due to increased levels in the overexpression the settings used for comparing the *UAS-RpS6* with the control were lower (Zeiss Meta settings, pinhole 1.2, gain 345). (C) Confocal images of 3^rd^ instar eye-antennal imaginal disc (top panel) and wing imaginal disc (bottom panel) at day 5 stained for anti-RpS6 antibody and DNA, genotypes marked.
(TIF)

Figure S4 *P0206-Gal4* driven reduction of *RpL38* by RNAi also results in small PGs and a larger wing disc. (A) Light micrographs of 3^rd^ instar larvae with genotypes indicated at day 5 for control and day 10 for the *UAS-RpS6* RNAi and *UAS-RpL38* RNAi. Confocal images of 3^rd^ instar prothoracic glands at (day 5 for control and day 10 for *UAS-RpL38* RNAi) stained for DNA and marked by co-expressing CD8-GFP. Magnification 40×. Scale bar 50 µM. (B) Fluorescent images of 3^rd^ instar wing discs (day 5 for control and day 10 for *UAS-RpL38* RNAi) stained for DNA bearing the genotypes indicated. Magnification 20×. (C) Graph of average wing disc area. Results are represented as the mean +/− standard error. Statistical analysis applied: unpaired t-test, where * = p<0.05.
(TIF)

Figure S5 CycE, BrdU and PH3 analysis of eye discs from *Amn^C651^>Dp110^DN^* suppression of *cycE^JP^*. (A) Confocal images of 3^rd^ instar eye imaginal discs stained for CycE and DNA with genotypes as indicated. White boxes mark the band of cycE cells in the SMW. Images were taken at 40× magnification. Orientation of eye discs: anterior (left), posterior (right). Scale bar equals 50 µm. (B) Confocal images of BrdU incorporation in 3^rd^ instar eye imaginal discs also stained for and DNA with genotypes indicated. White boxes mark the band of S phase cells. Images were taken at 40× magnification. Orientation of eye discs: anterior (left), posterior (right). Scale bar equals 50 µm. (C) Confocal images of 3^rd^ instar eye imaginal discs stained for cells in the SMW (PH3) and DNA with genotypes as indicated. White boxes mark the band of cells in SMW. Images were taken at 40× magnification with 0.7× optical zoom. Orientation of eye discs: anterior (left), posterior (right). Scale bar equals 50 µm. (D) Graph quantifying the number of cells in the SMW. Results are represented as the mean +/− standard error.
(TIF)

Table S1 Reducing *RpS6* in different tissues by RNAi. A table of the different *Gal4* drivers used to induce knockdown of *RpS6* with *UAS-RpS6* RNAi, and the phenotypes observed at 25°C and 18°C. Drivers used: *Actin-Gal4 (Act-Gal4), Tubulin-Gal4 (Tub-Gal4), Daughterless-Gal4 (Da-Gal4), engrailed-Gal4 (En-Gal4), MS1096-Gal4, Patched-Gal4 (Ptc-Gal4), Glass Multimer Reporter-Gal4 (GMR-Gal4), Eyeless-Gal4 (Ey-Gal4)*. Abbreviations: 1^st^ instar larvae (L1), 2^nd^ instar larvae (L2), 3^rd^ instar larvae (L3). N/A – not tested.
(DOC)

Table S2 Effects of reducing *RpS13*, *RpL5*, *RpL30* and *RpL38* in different tissues. A comparison between *RpS6* RNAi phenotypes (at 25°C) with those from *RpS13*, *RpL5*, *RpL30* and *RpL38* RNAi with a range of GAL4 drivers including: *Daughterless-Gal4 (Da-Gal4), engrailed-Gal4 (En-Gal4), MS1096-Gal4, Patched-Gal4 (Ptc-Gal4), Glass Multimer Reporter-Gal4 (GMR-Gal4), Eyeless-Gal4 (Ey-Gal4)*. 1^st^ instar larvae (L1), 2^nd^ instar larvae (L2), 3^rd^ instar larvae (L3). N/A – not tested.
(DOC)

Table S3 Log rank test of developmental data. Log rank test as calculated by GraphPad Prism software of genotypes as indicated from Figure 3C.
(DOC)

Table S4 Log rank test of developmental data. Log rank test as calculated by GraphPad Prism software of genotypes as indicated from Figure 7D.
(DOC)
