# Supplementary figures and images for: Drosophila Ribosomal Protein Mutants Control Tissue Growth Non-Autonomously via Effects on the Prothoracic Gland and Ecdysone
Source: PLoS Genet. 2011 Dec 15;7(12):e1002408. doi: 10.1371/journal.pgen.1002408 (PMC3240600; doi:10.1371/journal.pgen.1002408)

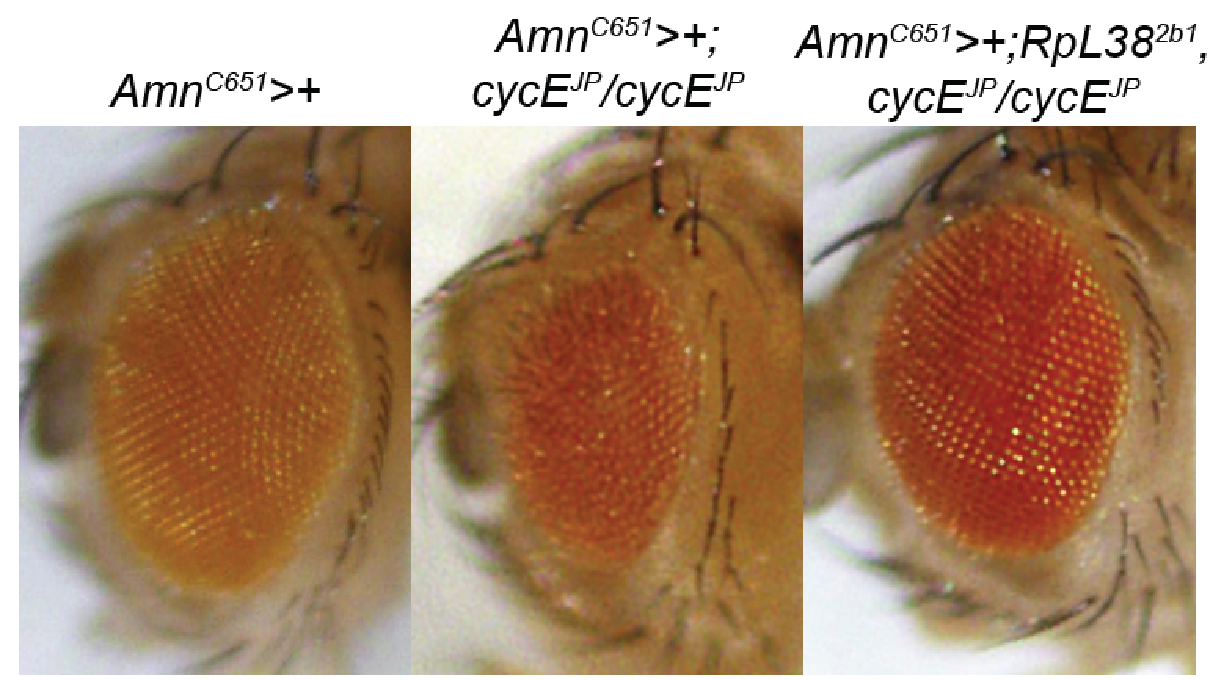

Supplement: Figure S1 — RpL382b1 suppresses cycEJP. Light micrographs of female adults bearing the genotypes indicated. (TIF) [file pgen.1002408.s001.tif]

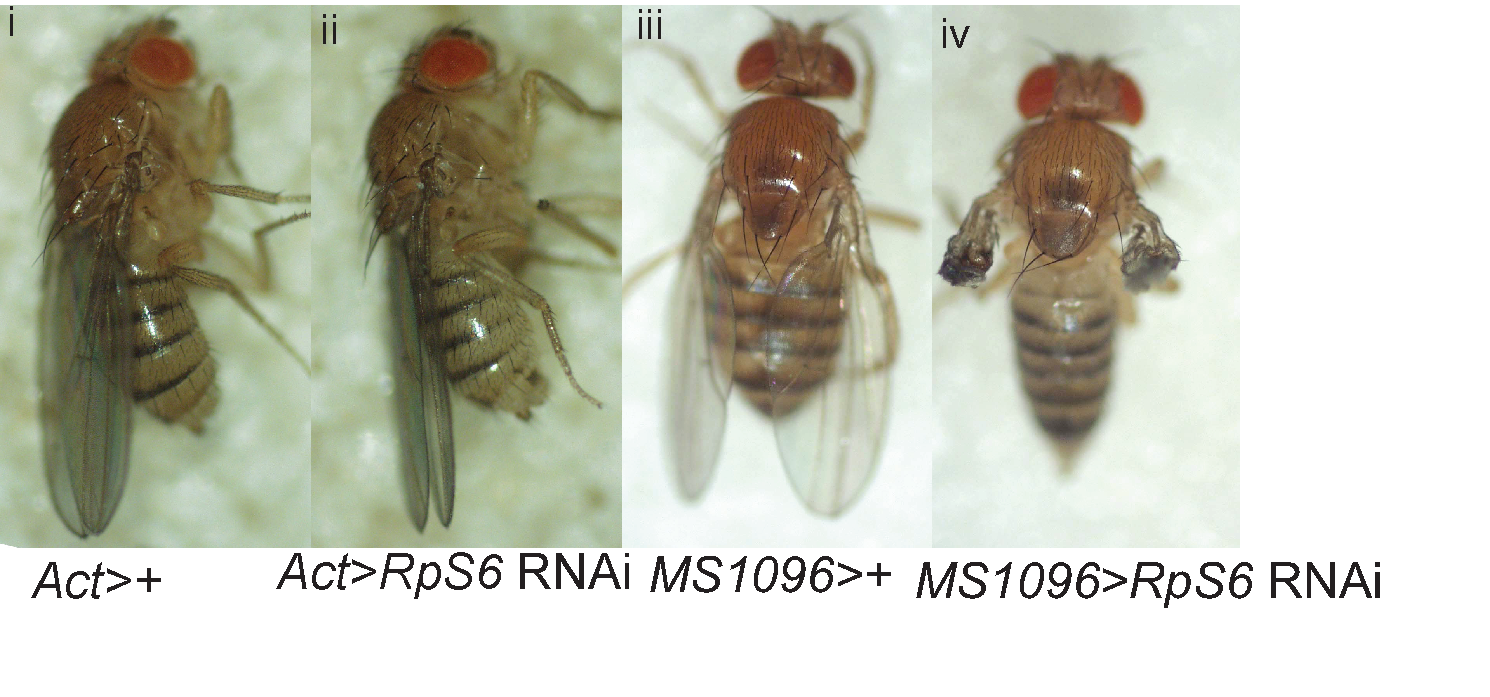

Supplement: Figure S2 — Reducing RpS6 in different tissues by RNAi. Light micrographs of female adults bearing the genotypes indicated. (TIF) [file pgen.1002408.s002.tif]

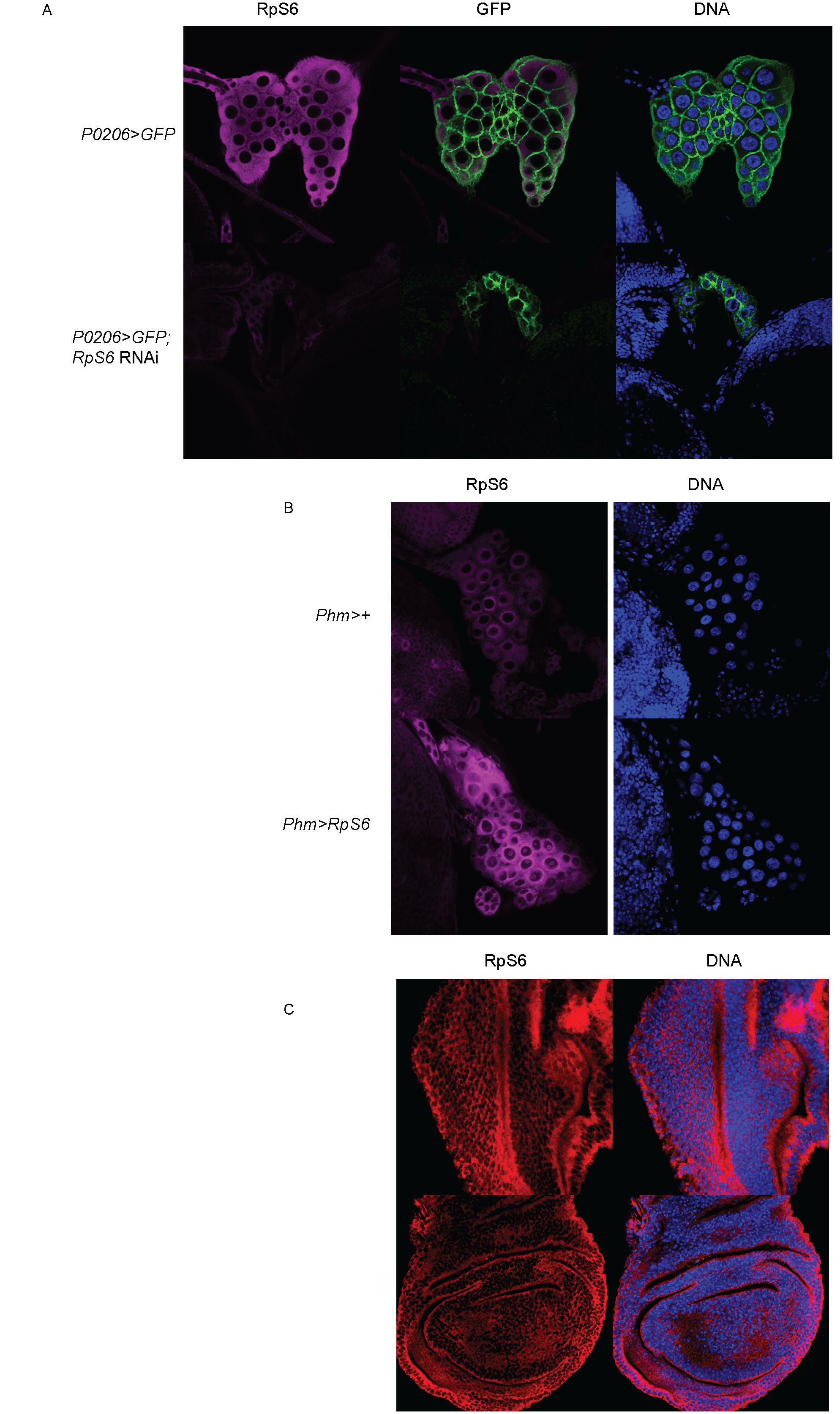

Supplement: Figure S3 — RpS6 protein is knocked down by UAS-RpS6 RNAi and overexpressed by UAS-RpS6. (A,B) Confocal images of 3rd instar prothoracic glands at day 5 stained for anti-RpS6 antibody and DNA, genotypes marked. P0206-Gal4 is a ring gland specific driver [42]–[43]. Phm-Gal4 is a PG specific driver [43]. Confocal images were taken at equivalent settings (Zeiss Meta settings, pinhole 1.2, gain 525) for comparison between the UAS-RpS6 RNAi and control. Due to increased levels in the overexpression the settings used for comparing the UAS-RpS6 with the control were lower (Zeiss Meta settings, pinhole 1.2, gain 345). (C) Confocal images of 3rd instar eye-antennal imaginal disc (top panel) and wing imaginal disc (bottom panel) at day 5 stained for anti-RpS6 antibody and DNA, genotypes marked. (TIF) [file pgen.1002408.s003.tif]

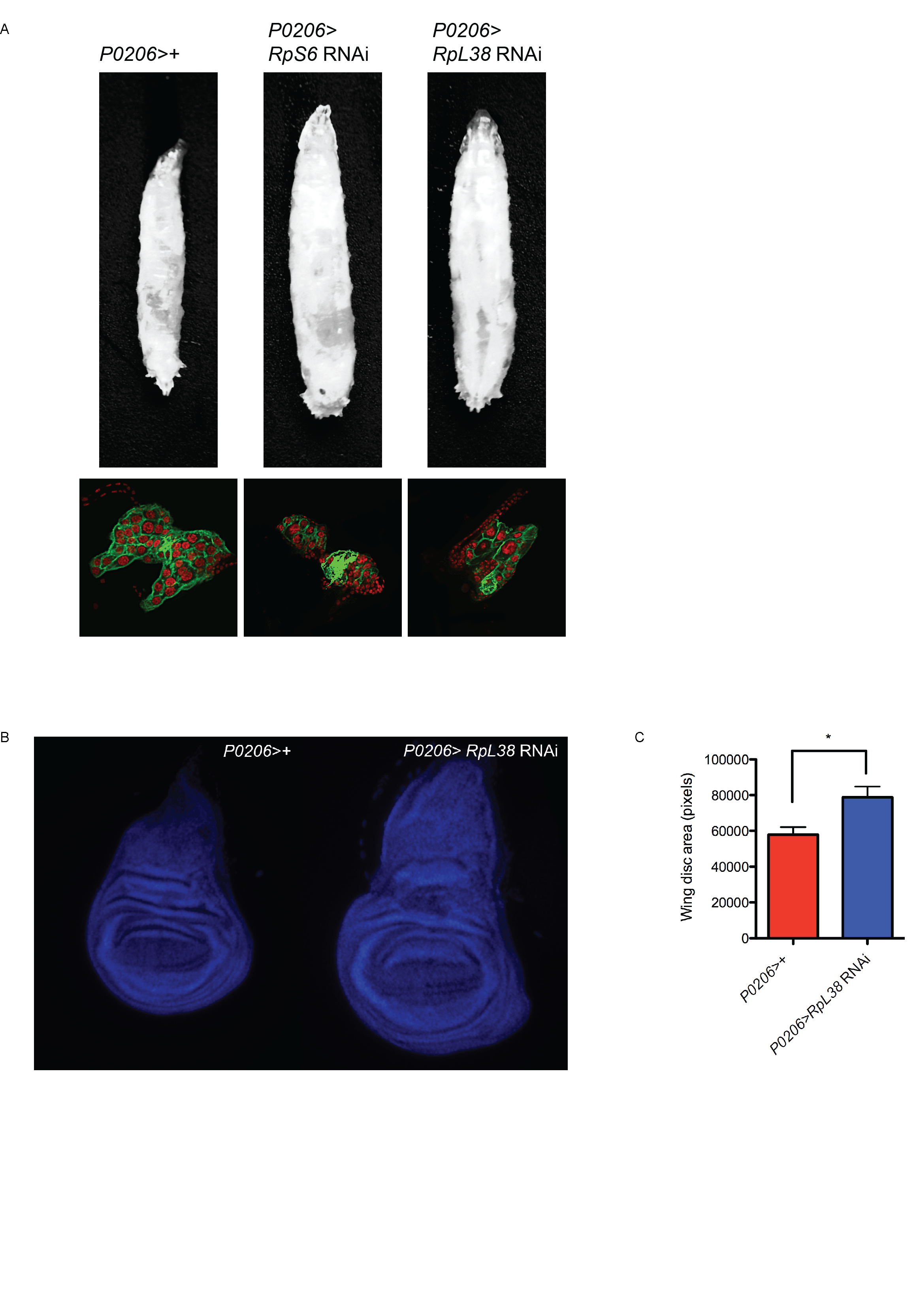

Supplement: Figure S4 — P0206-Gal4 driven reduction of RpL38 by RNAi also results in small PGs and a larger wing disc. (A) Light micrographs of 3rd instar larvae with genotypes indicated at day 5 for control and day 10 for the UAS-RpS6 RNAi and UAS-RpL38 RNAi. Confocal images of 3rd instar prothoracic glands at (day 5 for control and day 10 for UAS-RpL38 RNAi) stained for DNA and marked by co-expressing CD8-GFP. Magnification 40×. Scale bar 50 µM. (B) Fluorescent images of 3rd instar wing discs (day 5 for control and day 10 for UAS-RpL38 RNAi) stained for DNA bearing the genotypes indicated. Magnification 20×. (C) Graph of average wing disc area. Results are represented as the mean +/− standard error. Statistical analysis applied: unpaired t-test, where * = p<0.05. (TIF) [file pgen.1002408.s004.tif]

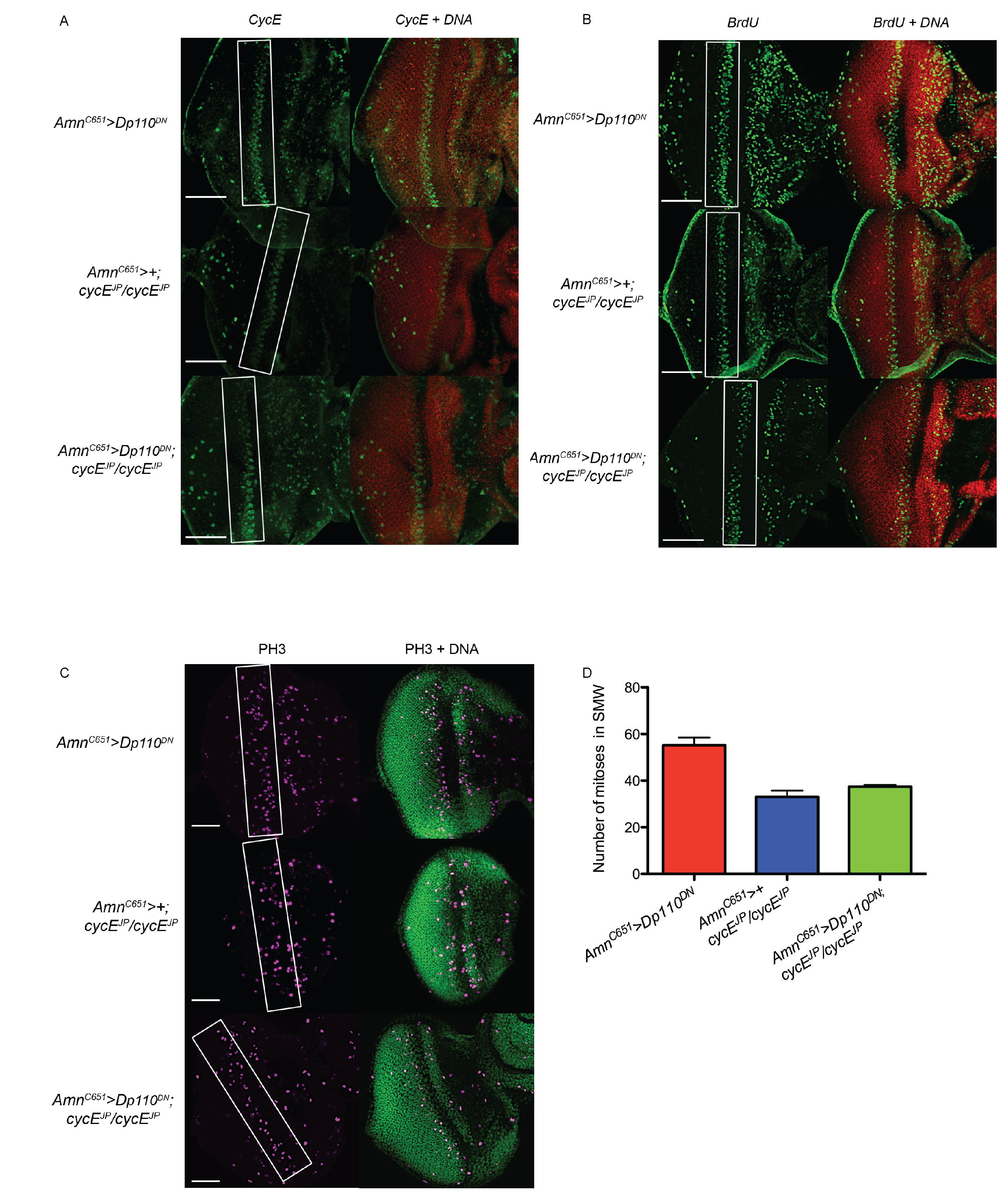

Supplement: Figure S5 — CycE, BrdU and PH3 analysis of eye discs from AmnC651>Dp110DN suppression of cycEJP. (A) Confocal images of 3rd instar eye imaginal discs stained for CycE and DNA with genotypes as indicated. White boxes mark the band of cycE cells in the SMW. Images were taken at 40× magnification. Orientation of eye discs: anterior (left), posterior (right). Scale bar equals 50 µm. (B) Confocal images of BrdU incorporation in 3rd instar eye imaginal discs also stained for and DNA with genotypes indicated. White boxes mark the band of S phase cells. Images were taken at 40× magnification. Orientation of eye discs: anterior (left), posterior (right). Scale bar equals 50 µm. (C) Confocal images of 3rd instar eye imaginal discs stained for cells in the SMW (PH3) and DNA with genotypes as indicated. White boxes mark the band of cells in SMW. Images were taken at 40× magnification with 0.7× optical zoom. Orientation of eye discs: anterior (left), posterior (right). Scale bar equals 50 µm. (D) Graph quantifying the number of cells in the SMW. Results are represented as the mean +/− standard error. (TIF) [file pgen.1002408.s005.tif]
