# Supplementary material for: Drosophila Ribosomal Protein Mutants Control Tissue Growth Non-Autonomously via Effects on the Prothoracic Gland and Ecdysone
Source: PLoS Genet. 2011 Dec 15;7(12):e1002408. doi: 10.1371/journal.pgen.1002408 (PMC3240600; doi:10.1371/journal.pgen.1002408)
Supplement: Table S1 — Reducing RpS6 in different tissues by RNAi. A table of the different Gal4 drivers used to induce knockdown of RpS6 with UAS-RpS6 RNAi, and the phenotypes observed at 25°C and 18°C. Drivers used: Actin-Gal4 (Act-Gal4), Tubulin-Gal4 (Tub-Gal4), Daughterless-Gal4 (Da-Gal4), engrailed-Gal4 (En-Gal4), MS1096-Gal4, Patched-Gal4 (Ptc-Gal4), Glass Multimer Reporter-Gal4 (GMR-Gal4), Eyeless-Gal4 (Ey-Gal4). Abbreviations: 1st instar larvae (L1), 2nd instar larvae (L2), 3rd instar larvae (L3). N/A – not tested. (DOC) [file pgen.1002408.s006.doc]

Supplementary Table 1 - Reducing *RpS6* in different tissues by RNAi.

| ***GAL4*** | **Expression pattern** | **Phenotype of *RpS6* reduction (25°C)** | **Phenotype of *RpS6* reduction (18°C)** |
| --- | --- | --- | --- |
| *Act-Gal4* | Weak, early, ubiquitous | Viable | Viable |
| *Tub-Gal4* | Strong, early, ubiquitous | Embryonic lethal | Embryonic lethal |
| *Da-Gal4* | Strong, early, ubiquitous | Larval (L1) lethal | Larval (L1) lethal |
| *En-Gal4* | Embryo – segment boundaries  Wing disc – posterior compartment | Larval (L1-3) lethal to Pupal lethal | Larval (L1-3) lethal to Pupal lethal |
| *MS1096-Gal4* | Wing disc – pouch compartment | Stumpy, shrivelled wings | Stumpy, shrivelled wings |
| *Ptc-Gal4* | Embryo  Wing disc – Anterior/Posterior boundary | Larval (L1-2) lethal | N/A |
| *GMR-Gal4* | Eye disc – posterior | Small, glassy, necrotic eyes | N/A |
| *Ey-Gal4* | Eye disc – early expression in all eye cells | Normal eyes | N/A |
