# Supplementary material for: Drosophila Ribosomal Protein Mutants Control Tissue Growth Non-Autonomously via Effects on the Prothoracic Gland and Ecdysone
Source: PLoS Genet. 2011 Dec 15;7(12):e1002408. doi: 10.1371/journal.pgen.1002408 (PMC3240600; doi:10.1371/journal.pgen.1002408)
Supplement: Table S2 — Effects of reducing RpS13, RpL5, RpL30 and RpL38 in different tissues. A comparison between RpS6 RNAi phenotypes (at 25°C) with those from RpS13, RpL5, RpL30 and RpL38 RNAi with a range of GAL4 drivers including: Daughterless-Gal4 (Da-Gal4), engrailed-Gal4 (En-Gal4), MS1096-Gal4, Patched-Gal4 (Ptc-Gal4), Glass Multimer Reporter-Gal4 (GMR-Gal4), Eyeless-Gal4 (Ey-Gal4). 1st instar larvae (L1), 2nd instar larvae (L2), 3rd instar larvae (L3). N/A – not tested. (DOC) [file pgen.1002408.s007.doc]

Supplementary Table 2 - Effects of reducing different *Rp* RNAi in different tissues.

| ***UAS-Rp* RNAi** | ***Da-Gal4*** | ***En-Gal4*** | ***MS1096-Gal4*** | ***Ptc-Gal4*** | ***GMR-Gal4*** | ***Ey-Gal4*** |
| --- | --- | --- | --- | --- | --- | --- |
|  | Early, ubiquitous | Embryo – segment boundaries  Wing disc – posterior compartment | Wing disc – pouch compartment | Embryo  Wing disc – Anterior/Posterior boundary | Eye disc – posterior | Eye disc – early expression in all eye cells |
| ***RpS6*** | Larval (L1) lethal | Larval (L1-3) lethal to Pupal lethal | Stumpy, shrivelled wings | Larval (L1-2) lethal | Small, glassy, necrotic eyes | Normal eyes |
| ***RpS13*** | Lethal | Lethal | Small/stubby wings, Minute bristles | N/A | Small, glassy eye with necrotic patches | N/A |
| ***RpL5*** | Lethal | Lethal | Small wings | N/A | Small, glassy eyes often with necrotic patches | N/A |
| ***RpL30*** | Lethal | Lethal | Small curled up wings, weak Minute bristles | N/A | Glassy eyes, some necrotic patches | N/A |
| ***RpL38*** | Lethal | Lethal | Small wings, Minute bristles | N/A | Slightly small glassy eye, rare necrotic patches | N/A |
