# Supplementary material for: Drosophila Ribosomal Protein Mutants Control Tissue Growth Non-Autonomously via Effects on the Prothoracic Gland and Ecdysone
Source: PLoS Genet. 2011 Dec 15;7(12):e1002408. doi: 10.1371/journal.pgen.1002408 (PMC3240600; doi:10.1371/journal.pgen.1002408)
Supplement: Table S3 — Log rank test of developmental data. Log rank test as calculated by GraphPad Prism software of genotypes as indicated from Figure 3C. (DOC) [file pgen.1002408.s008.doc]

Supplementary Table 3 – Log-rank test of developmental delay data of Figure 3C

| **Genotypes compared** | **Log-rank: Chi-Square** | **df** | **Log-rank: Chi-Square *p*** |
| --- | --- | --- | --- |
| *RpS6^WG1288^/Amn^C651^* -20E vs +20E | 5.104 | 1 | 0.0239 |
| *RpS6^WG1288^/Amn^C651^; cycE^JP^/ cycE^JP^* -20E vs +20E | 3.021 | 1 | 0.0822 |
