# Supplementary material for: Drosophila Ribosomal Protein Mutants Control Tissue Growth Non-Autonomously via Effects on the Prothoracic Gland and Ecdysone
Source: PLoS Genet. 2011 Dec 15;7(12):e1002408. doi: 10.1371/journal.pgen.1002408 (PMC3240600; doi:10.1371/journal.pgen.1002408)
Supplement: Table S4 — Log rank test of developmental data. Log rank test as calculated by GraphPad Prism software of genotypes as indicated from Figure 7D. (DOC) [file pgen.1002408.s009.doc]

Supplementary Table 4 – Log-rank test of developmental delay data of Figure 7D

| **Genotypes compared** | **Log-rank: Chi-Square** | **df** | **Log-rank: Chi-Square *p*** |
| --- | --- | --- | --- |
| *RpS6^WG1288^/+;;P0206>+*vs *RpS6^WG1288^/+;;P0206>RpS6* | 5.629 | 1 | 0.0177 |
